# Analysis of the distribution of health services in the State of São Paulo: focus on Big Data

**DOI:** 10.31744/einstein_journal/2025AO1070

**Published:** 2025-07-11

**Authors:** Márcio Alexandre Marques, Caio Fernandes Chaves Maximiano, Thiago Gonçalves dos Santos Martins, Augusto Voltaire do Nascimento

**Affiliations:** 1 Instituto de Ciência e Tecnologia, Engenharia de Controle e Automação Universidade Estadual Paulista “Júlio de Mesquita Filho” Sorocaba SP Brazil Instituto de Ciência e Tecnologia, Engenharia de Controle e Automação, Universidade Estadual Paulista “Júlio de Mesquita Filho”, Sorocaba, SP, Brazil.; 2 Instituto de Ciências Médicas Universidade Federal do Rio de Janeiro Macaé RJ Brazil Instituto de Ciências Médicas, Universidade Federal do Rio de Janeiro, Macaé, RJ, Brazil.; 3 Hospital do Servidor Público Estadual Francisco Morato de Oliveira São Paulo SP Brazil Hospital do Servidor Público Estadual Francisco Morato de Oliveira, São Paulo, SP, Brazil.

**Keywords:** Big Data, Health services accessibility, Geographic locations, Health facilities, Health personnel, Power BI, Dashboard systems, Apache Spark, CNES, PostgreSQL, Database, Public health

## Abstract

**Objective:**

To develop a process (pipeline) for extracting, processing, and analyzing data from the National Registry of Health Establishments in the State of São Paulo, Brazil, to evaluate the distributions of health professionals and services throughout this state.

**Methods:**

Big Data resources were used to acquire, process, and aggregate health-related information, with the creation of a relational database for the local storage of processed data. The entire process is implemented using a framework that enables the creation and editing of a series of instructions for performing specific tasks (scripts) in different languages. For the end user interface, an interactive panel of information, metrics, graphs, and numerical indicators was created to show the distribution of health professionals and services across the municipalities of the State of São Paulo.

**Results:**

The developed tool generates a process for monthly updating of a database, producing a dynamic information report that allows users to perform queries on quantitative and qualitative indicators, providing a general overview of health establishments, and furnishing information on specific health professionals.

**Conclusion:**

The preliminary results indicated that the developed tool is potentially scalable and could contribute to the identification of regions of the state requiring action from public authorities, helping to optimize the hiring of doctors and community health agents, among others.

## In Brief

A tool was developed to extract, process, and analyze data from the National Registry of Health Establishments of São Paulo, creating a relational database and an interactive panel. This tool enables queries about health professionals and services, generating dynamic reports that assist in the optimized allocation of resources and public management.

## Highlights

■ The extract, transform, and load algorithm automates data extraction and updates in the PostgreSQL database.

■ The interactive dashboard enables detailed queries on establishments and professionals.

■ Results highlight medical doctor deficit in 22 cities and a shortage of community agents.

■ The tool can scale and assist managers in resource allocation for healthcare.

## Graphical abstract

**Figure:**
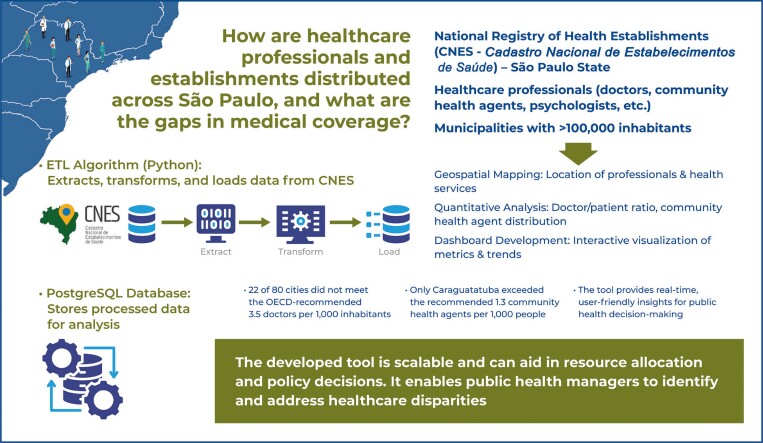

## INTRODUCTION

In countries with continental dimensions, such as Brazil, managing the services provided to the population poses major challenges. Even without considering resource allocation, there is a lack of interest by qualified labor in providing basic services in the health sector in regions far from large urban centers. The mismatch between demand and supply of doctors in Brazil is related to a lack of resources and structures for working in smaller cities, salary levels, and other issues.^1^

Scheffer et al.^4^ reported that between 2015 and 2020, the Brazilian population increased by 4.8%, while the number of doctors increased by 25.1% due to expansion and greater availability of courses and undergraduate programs in medicine. Although there have been changes in the demographics of this profession, its distribution across Brazil remains unequal, even after the initial impact of the quantitative increase in healthcare professionals arising from the growing number of medical schools in the last decade.^5^

Although São Paulo is the state with the most resources in the country, analysis shows an evident lack of homogeneity in the distribution of health professionals and services across the different regions, with the data indicating that the capital city (São Paulo) has 2.4 times more doctors per inhabitant than the interior regions of the state. This situation has repercussions on the population in cases of medical emergencies due to the dependence on public transport to visit the nearest medical professional or even the lack of basic services, such as vaccinations and complementary medical examinations.^6,7^

Against this background, the present study involved using technology to process large amounts of data (Big Data)^8^ by developing a tool that employs the information made available on the National Registry of Health Establishments (CNES - *Cadastro Nacional de Estabelecimentos de Saúde*) platform^13^ to evaluate the distribution of health professionals and services throughout the State of São Paulo, aiming to assist in ensuring a more rational use of resources.

The developed application provides a tool to investigate the lack of certain health services and professionals in a given region, dynamically describing the situation and highlighting bottlenecks in public management. This can assist decision-making by health managers in public and private institutions, with the creation of incentives that could mitigate the identified deficiencies and improve the quality and delivery of services offered to the population.

During the first stage of the tool development, there was no discussion with public health authorities because the scope of the work was restricted to the treatment and aggregation of data collected from the CNES, enabling the generation of an interactive information panel for the analysis and visualization of data by the user. In the next stage, health managers were involved to evaluate and suggest improvements, as the aim was to make the application widely available for routine use by health services in the State of São Paulo.

## OBJECTIVE

To develop a data pipeline to collect and process information made available on the National Registry of Health Establishments platform, making it possible to use dashboards to dynamically visualize, analyze, and evaluate the distribution of health system professionals and services throughout the State of São Paulo.

## METHODS

### Extract, transform, and load

As described by Mahmood,^17^ the extract, transform, and load (ETL) process involves moving a massive amount of data at three different levels, proceeding from one or more sources toward a destination. Owing to the volume of information and speed of its acquisition, the real-time ETL process is performed by extracting and transforming the flow of unstructured data from multiple sources in dispersed environments, with the overall technique being directed toward the provision of important and relevant results that can be analyzed and worked on.^18,19^

### Big Data

According to Taurion,^11^ Big Data refers to a set of technologies, processes, and practices that allow users to analyze previously inaccessible information, consequently facilitating decision making and efficient management of activities.

This concept involves the use of tools capable of analyzing large or complex datasets and systematically evaluating the information extracted from various sources. Currently, there are many questions related to the privacy, ethics, and security of information management. Nevertheless, the concept is being used in the health sector for data analysis, outcome prediction, and achieving improvement, assisting health professionals in precise decision-making as well as disease diagnosis and prediction.^20^

Big Data concerns large volumes of data and can be exemplified by the five Vs: volume (of data), variety (of sources), velocity (of processing), veracity (reliability), and value (values obtained).^12^

**Figure 1:**
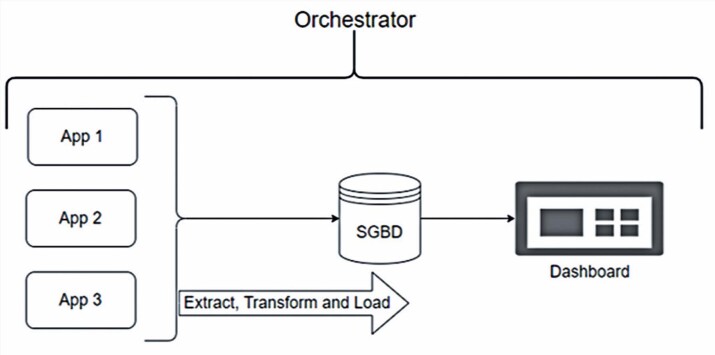
Example of data architecture for performing extract, transform, and load

The increase in the volume of information in recent years has led to the development of a wide range of Big Data tools in addition to sets of models and rules for managing and controlling data. Figure 1 shows an approach involving the integration of different applications, where each web application (Apps 1, App 2, and App 3) is responsible for a certain task. The obtained information can be stored in a database management system (DBMS) for subsequent use.

### Apache Spark

Apache Spark is an open-source distributed processing tool for real-time Big Data processes that uses in-memory processing support to increase the performance of queries for large volumes of data compared to on-disk processing.^23^

It has several favorable characteristics for processing and storing large sets of information.

Integration: this adds complex and relevant features, such as graph algorithms and reinforcement learning, making it a robust solution for various applications that use data.

Speed: one of the main characteristics of Spark is that it addresses the five Vs of Big Data via its in-memory processing, providing fast solutions when compared to other techniques for processing information.

Flexibility: spark supports multiple languages, and allows developers to create applications in Java, Scala, R, and Python.

Data streaming: spark streaming facilitates the creation of fault-tolerant processing flows using real-time data.

### PostgreSQL

PostgreSQL is an open-source, highly stable DBMS that supports different structured query language (SQL) functions, such as foreign keys, subqueries, triggers, and different user-defined types and functions. It augments the SQL by offering several features that meticulously scale and reserve data workloads. It is primarily used to store information for mobile, Web, geospatial, and analytical applications.^26, 27^

Because of its easy integration with different company tools and systems, PostgreSQL has become one of the most popular data storage solutions in the market. The main features of this tool include multiversion concurrency control, point-in-time recovery, tablespaces, asynchronous replication, grouped transactions, sophisticated query planner (optimizer), and sequential transaction logger for fault tolerance.^28^

The pgAdmin graphical DBMS administration software, which provides a user-friendly graphical interface, can be used to develop queries and create objects using PostgreSQL.

### Power business sense (BI)

Power BI is software designed to transform data into information for BI. This tool allows users to connect to a wide variety of sources (including txt, Excel, csv, databases, and websites) to extract data, generate performance indicators, and create dashboards.^29^

Power BI enables the creation of information models that are related to different tables and enable the processing and transformation of data from highly disparate sources. The dynamic representation of data source columns makes it possible to generate a variety of dashboards and indicators, in addition to enabling publication on the internet so that different people can have access to the developed project.^30^

### Database

The Brazilian CNES constitutes the database for creating an operational health information system that legally requires registering all health establishments, whether public, third-party, or private as well as individuals and legal entities that perform any type of healthcare service within the country. The CNES emerged from efforts to reformulate the previously used registration forms, defining registration requirements for all mobile and hospital health facilities. The aim is to provide detailed data that more closely represent the reality of the country, states, and municipalities regarding the provision of health services.^31^

In addition, this system allows the automation of all data collection processes performed by states and municipalities, including the installed physical capacity, services available, and professionals associated with health establishments and family health teams, providing health managers with national data. This enables the mapping of all health institutions, ensuring the legality of their operations, and facilitating the search for specialized services, as information is available concerning health teams, professionals, and facilities, considering aspects such as infrastructure, available beds, and type of service provided.^31^

Given the features described above, the CNES database was used as the data source because it is an easily accessed national public platform that is updated monthly.^13,31^ It integrates mandatory information from all health units, covering an extensive set of indicators, including the number of health facilities in each municipality and public contracts of doctors.^15,16^

### Data architecture

**Figure 2:**
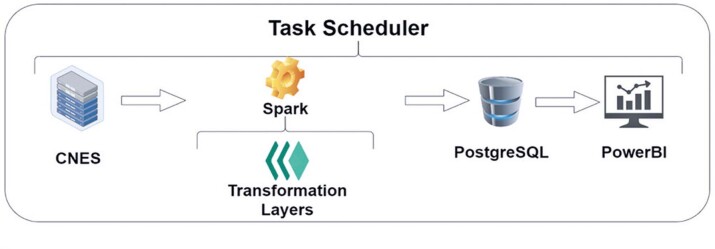
Extract csv files from zip files

An architecture was developed to perform the ETL process, targeting three main aspects: data storage, processing, and visualization, as shown in figure 2. In this architecture, the CNES server is the source of the initial data extraction.

The data transformation employed the Spark framework for data cleaning and aggregation, together with local folders that functioned as layers. After the first two steps, the final data were sent to the PostgreSQL database, which constituted a storage platform and source of information for developing the reports using the Power BI software.

### Data visualization: interface and functionalities

The data were visualized using Power BI, where a single dashboard can aggregate different methods of analysis, such as clustered column and line charts, choropleth maps, and filters, making the user experience much more intuitive and enabling the generation of value from the raw data.

Different indicators were also created using the DAX (Data Analysis Expressions) language provided by the tool itself. To highlight the information most important to the user, a form of visualization called “card” was employed, consisting of a single piece of easily observed information that was generally placed alongside other relevant data.

The developed interface allows the insertion of filters into the dashboard, enabling navigation through different user-defined scenarios and making the experience more interactive and complete.

### Extract, transform, and load process

To develop the ETL process, Spark framework commands were used to process the CNES data. Three auxiliary layers were defined for the ETL processes.

- ZIP_FILES: in the first layer, compressed files from the data platform are stored in their “raw” form.

- CSV_FILES: the second layer contains data in “csv” format (after being extracted from the compressed file), serving as a repository for aggregations.

CURATED_FILES: the third layer contains data available for use and delivery to the PostgreSQL database.

**Figure 3:**
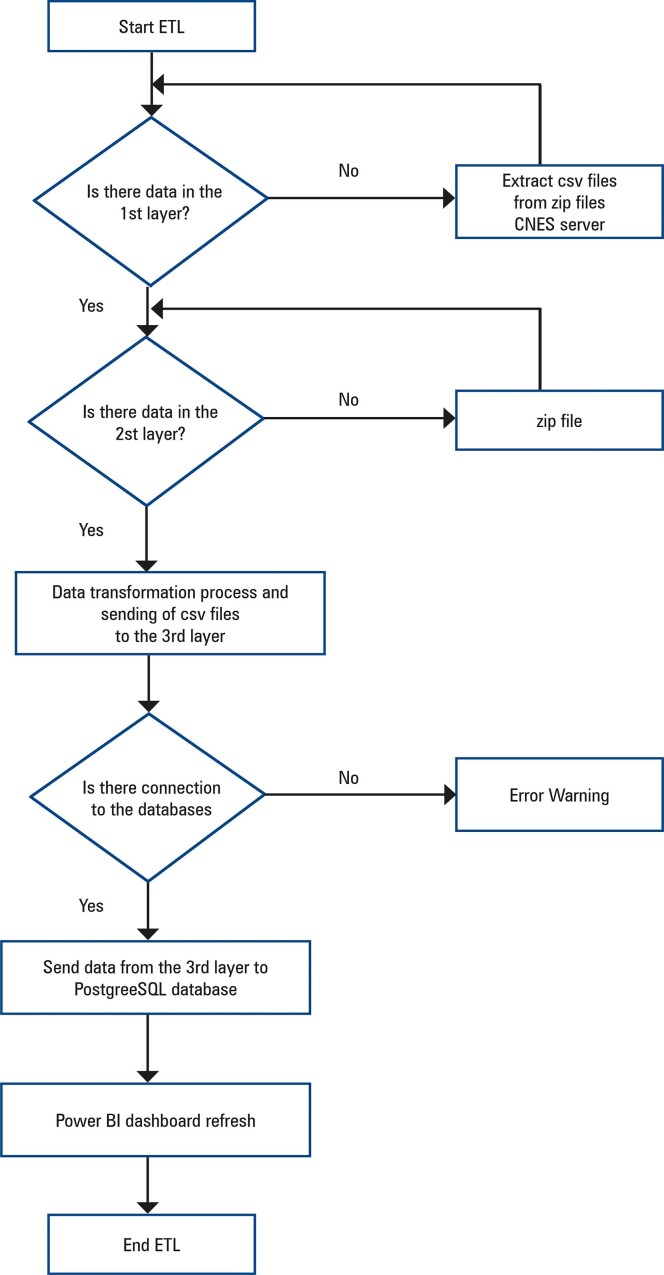
Extract, transform, and load process diagram

Figure 3 illustrates how the layers contribute to the ETL process, from the extraction of data from the CNES platform to insertion into the relational database and updating of the Power BI.

ETL: extract, transform, and load.

In this process, all the steps of extraction, transformation, and loading of notebooks were combined into a single Python file responsible for initializing the libraries and functions. Hence, a single Python script could be used to execute the end-to-end process, with update of the tables in the PostgreSQL database, and completing a stage, with update of the report in Power BI.

### Windows task scheduler

After the ETL and creating the dashboard for a certain period, it was necessary to automate the process, such that the dataset could be automatically updated without manual execution. This was achieved using the Windows Task Scheduler tool, a feature of Microsoft’s operating system. This tool makes it possible to perform predefined activities that are executed automatically when a certain set of conditions is met (*e.g*., day/time or the arrival of a file in a certain directory).

There are several methods to trigger the automatic execution of an action, resulting in the creation of a task that calls an external service, such as the execution of the script responsible for carrying out the ETL process.

In the case of the tool presented here, this process was developed, with the aim of using data from two months prior to the execution date. Therefore, the task was configured to be conducted on the 7^th^ of each month, allowing time for data to be made available on the CNES platform.

Once the script execution action has been identified and configured for the periods in which it must be processed, updating the database no longer requires manual intervention by the user because the entire ETL process is included in the script automatically by the Windows Task Scheduler.

### Data validation

Ensuring the quality and integrity of the data is a central aspect of the developed ETL process because the metrics were calculated based on the data extracted from the CNES platform. Various validation and error treatment techniques were applied to ensure the reliability of the obtained information.

During data extraction, the system checks for the presence of. zip files in the destination directory before starting the downloads (first layer) to avoid unnecessary duplication and to optimize storage.

In the transformation phase, the Spark framework validates the structure and format of the csv data (second layer), with essential fields being included using PostgreSQL and restrictions such as “not null” and integrity of the registry keys (SK_REGISTRO). Treatment of exceptions with “try-except” blocks identifies problems, such as download failures or data inconsistencies, ensuring clear messages, continuous execution, generation of program execution logs, and integrity for resolution of any problem. Temporary files (such as. zip files) were removed after processing to avoid redundancy.

Finally, automation using the Windows Task Scheduler ensures consistent updates, while the layered architecture consisting of .zip files, .csv files, and curated files (third layer) provide a robust structure and allow partial reprocessing when necessary. This approach ensures the provision of reliable data for the dashboards.

### Equipment

To implement the tool described in this article and execute the entire data pipeline, an HP Elitebook notebook computer with the following configuration: Intel Core i7 processor, 32 GB of RAM, and Windows 11 operating system was used.

The algorithm was implemented in the Anaconda IDE programming environment, which is a data science platform for Python, in conjunction with Jupyter Notebook, a free open-source application that provides a graphical interface for editing notebooks in a web browser.

## RESULTS

### Extract, transform, and load and dashboard process

**Figure 4:**
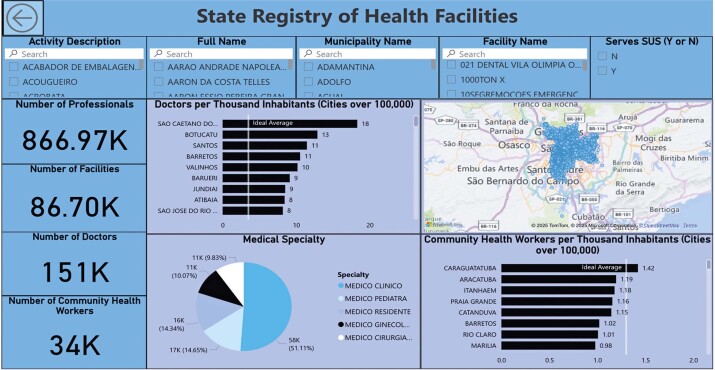
Dashboard developed using Power BI

The entire ETL process and the use of Power BI with PostgreSQL tables were executed automatically with the development of a dashboard to act as a user interface, enabling the use of filters and graphs according to the application desired by the user (Figure 4).

**Figure 5:**
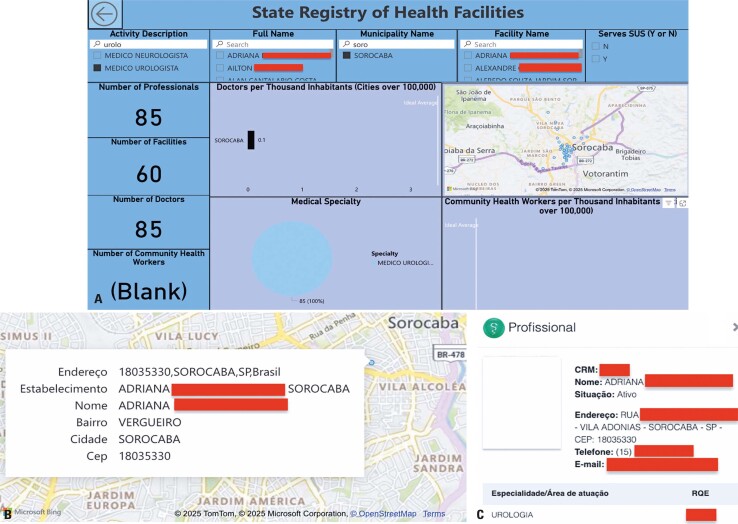
(A) Test scenario obtained using filters; (B) map with information; and (C) checking the selected doctor

As a test scenario, urologists in Sorocaba were searched using filters located at the top of the dashboard. Once the options were selected according to the desired context, a list was generated with the names of the professionals who met this requirement (Figure 5A). The test resulted in a list of 85 urologists in Sorocaba distributed across 60 establishments, with one doctor selected to confirm the data regarding the services provided. Figure 5B shows the information on the selected professionals and their establishments.

All the values collected from the Power BI map were validated using the Medical Guide of the São Paulo Regional Medicine Council webpage^32^ to check the test scenario (Figure 5C). This research simulation was carried out, as an example, by a user who needed to find a place for treatment by the specialist in question.

### Quantitative and qualitative indicators

Using the DAX language, the quantitative values collected were presented using a card, highlighting the main required numerical information and facilitating the analysis of the dashboard. In this scenario (Figure 4), the quantitative indicators were 867,000 health professionals, 87,000 health establishments, 151,000 doctors, and 34,000 community health agents.

It was also possible to obtain data for the main medical specialties available in the State of São Paulo as well as for general practitioners. Table 1 presents the distribution of doctors in the State of São Paulo.

**Table 1:** Distribution of doctors in the State of São Paulo

Medical specialty	Quantity (%)
General practice	58,000 (51.11)
Pediatrics	17,000 (14.65)
Residencial	16,000 (14.34)
Gynecology	11,000 (10.07)
General surgery	11,000 (9.83)

The tool also provided a qualitative indicator of the availability of doctors in cities with more than 100,000 inhabitants, which generally act as large centers serving neighboring and smaller cities.

Table 2 shows the grouping of cities at the top of this ranking that also have a good human development index or medical schools.

**Table 2:** Availability of doctors per 100 thousand inhabitants

City	Number of doctors per 100 thousand inhabitants
São Caetano do Sul	18
Botucatu	13
Santos	11
Barretos	11
Valinhos	10
Barueri	9
Jundiaí	9
Atibaia	8
São José do Rio Preto	8

The tool also provided a qualitative indicator of the numbers of community health agents in cities with more than 100,000 inhabitants, with four cities having the highest numbers: Caraguatatuba, Araçatuba, Itanhaém, and Praia Grande, with 1.42, 1.19, 1.18, and 1.16 community health agents per thousand inhabitants, respectively.

### User experience

During the development of the tool, evaluations of usability and efficiency were performed by two doctors and two information technology professionals, who tested the software to confirm its satisfactory performance and optimize the user experience. The tests addressed all aspects of interaction with the system, including user contact with the interface, ease of navigation, loading speed, and relevant content. The evaluations were performed at different phases of the project and included virtual and in-person sessions, with the participants exploring the platform and sharing their opinions and detailed information regarding its usability and efficiency.

The main suggestions concerned changes and reorganization of the interface and presentation of graphs. Based on the observations and suggestions of the medical doctors, the arrangement of the filters and indicators was adjusted to facilitate navigation and highlight critical information. The graphs were changed to more intuitive formats with clearer legends and better organization. These adjustments enhanced the visualization, enabling faster and more accurate analyses. Thus, the interactions between the users of the system and its developers led to the incorporation of all the suggested improvements into the tool.

## DISCUSSION

The ETL process algorithm for extracting, treating, and analyzing data from the CNES of the State of São Paulo is a strong point of this study. Using a single Python script, it automatically executes the entire process from end to end, subsequently updating all tables in the PostgreSQL database.

The study showed that the developed dashboard is fully interactive, allowing the user to navigate the data in any desired way, such as checking information on health establishments and professionals according to the region or medical specialty or even searching for a particular medical professional.

Figure 5C shows that the service location of the specialist chosen in the test was consistent with the location shown on the dashboard, considering both the address provided and the zip code (Figure 5B), indicating that the information provided by the tool was reliable, as it was consistent with that in the Medical Guide of the São Paulo Regional Medicine Council. This result also highlights the consistency of data transformation between the different files utilized during the ETL process.

Another highlight of the tool is that it allows the user to obtain quantitative and qualitative information about the distribution of healthcare professionals and establishments (Table 1), which is automatically updated as the ETL process is executed on the 7^th^ of every month.

Table 1 shows a predominance of general practitioners in the state, as these often provide primary care to the population in health centers.

According to the Organization for Economic Cooperation and Development, the ideal number of doctors per thousand inhabitants should be 3.5, a value reached in many cities in the State of São Paulo (Table 2), which is the richest state in the country and has the highest number of doctors.^33^

According to Ordinance No. 2,488, of October 2, 2011, published by the Ministry of Health, the number of community health agents covering 100% of the population should be equivalent to 1 for every 750 people.^34^ Therefore, the minimum value should be 1.3 for each group of 1000 people.

The results showed that for cities with a population above 100,000, only the city of Caraguatatuba presented a value of 1.3 community health agents per thousand inhabitants. This highlights the need for public authorities to create and maintain community health agent positions to ensure quality of life and healthcare for citizens.

Given the dynamic nature of the dashboard, in addition to the results presented here, this tool allows the user to produce other dashboards, according to specific needs. For example, in a particular city, it is possible to determine whether a certain professional works in the Brazilian Unified Health System (SUS - *Sistema Único de Saúde*), as well as the service location (and the most appropriate location, if the person works in more than one place). Another interesting feature of the tool is the possibility of searching for psychologists or psychopedagogues in the city in question, showing that it has a wide applicability for analyzing different scenarios in the health sector.

It should be noted that during the design and development process of the tool, priority was given to usability and user experience, and thus improvements suggested by the users not only made the tool more practical and effective but also enabled it to be adapted to their needs.

Therefore, the contact of users with the system interface made the tool easy to access, simple to learn, and intuitive to use, enabling tasks to be performed efficiently and in real time, with the changes made reinforcing its potential to support strategic decision-making in the public health sector. In the next stage, the tool will be submitted for analysis by public health managers to confirm its utility, adaptability, and effectiveness.

## CONCLUSION

The results obtained using the developed extract, transform, and load process algorithm and data from the National Registry of Health Establishments of the State of São Paulo showed that this tool can provide qualitative and quantitative indicators for health professionals and establishments. The results highlighted that 22 of the 80 cities with more than one hundred thousand inhabitants did not reach the minimum value of 3.5 doctors per thousand inhabitants recommended by the Organization for Economic Cooperation and Development. Similarly, regarding the distribution of community health agents, only the city of Caraguatatuba exceeded the value of 1.3 per thousand inhabitants recommended by the Ministry of Health.

The tool developed herein could be used to create an interactive panel, enabling adaptation to individual needs, navigation through different scenarios, and assisting in analyzing and understanding the real needs of municipalities in the State of São Paulo. Assessing the scalability potential of the tool in future studies could enable the provision of information needed by public authorities to identify any deficiencies in the health sector, thereby contributing to the improvement of health services in the State of São Paulo.
